# Clinical Applications and Future Directions of Minimal Residual Disease Testing in Multiple Myeloma

**DOI:** 10.3389/fonc.2020.00001

**Published:** 2020-01-31

**Authors:** Stefania Oliva, Mattia D'Agostino, Mario Boccadoro, Alessandra Larocca

**Affiliations:** Myeloma Unit, Division of Hematology, University of Torino, Azienda Ospedaliero-Universitaria Cittá Della Salute e Della Scienza di Torino, Turin, Italy

**Keywords:** multiple myeloma (MM), minimal residual disease (MRD), clinical practice, next-generation flow (NGF), next-generation sequencing (NGS), PET/CT

## Abstract

In the last years, the life expectancy of multiple myeloma (MM) patients has substantially improved thanks to the availability of many new drugs. Our ability to induce deep responses has improved as well, and the treatment goal in patients tolerating treatment moved from the delay of progression to the induction of the deepest possible response. As a result of these advances, a great scientific effort has been made to redefine response monitoring, resulting in the development and validation of high-sensitivity techniques to detect minimal residual disease (MRD). In 2016, the International Myeloma Working Group (IMWG) updated MM response categories defining MRD-negative responses both in the bone marrow (assessed by next-generation flow cytometry or next-generation sequencing) and outside the bone marrow. MRD is an important factor independently predicting prognosis during MM treatment. Moreover, using novel combination therapies, MRD-negative status can be achieved in a fairly high percentage of patients. However, many questions regarding the clinical use of MRD status remain unanswered. MRD monitoring can guide treatment intensity, although well-designed clinical trials are needed to demonstrate this potential. This mini-review will focus on currently available techniques and data on MRD testing and their potential future applications.

## Introduction

The treatment course of multiple myeloma (MM) has been strongly improved during the last 20 years: the introduction of modern 3-drug regimen therapies combined with transplantation increased the achievement of deeper responses and the acquisition of minimal residual disease (MRD) negativity in up to 40/50% of patients enrolled in clinical trials (1). Consistently, a large number of studies showed that, among patients achieving a complete response (CR), those with detectable MRD had inferior progression-free survival (PFS), and overall survival (OS) compared to those with undetectable MRD. Moreover, among patients in CR, improved PFS and OS have been significantly associated with undetectable MRD, regardless of disease stage, prior transplant, or cytogenetic risk (2).

**Figure 1:**
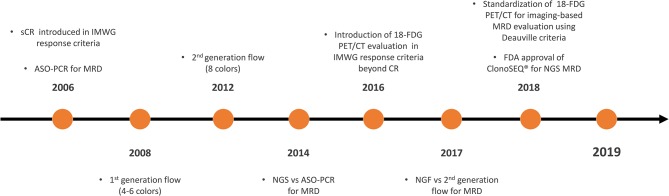
Timeline on the development and validation of MRD techniques in MM. MRD, minimal residual disease; MM, multiple myeloma; CR, complete response; sCR, stringent CR; IMWG, International Myeloma Working Group; ASO-PCR, allele-specific oligonucleotide polymerase chain reaction; NGS, next-generation sequencing; 18-FDG PET/CT, 18-fluorodeoxyglucose positron emission tomography/computed tomography; ClonoSEQ®, ClonoSEQ® Assay (Adaptive Biotechnologies, Seattle, US-WA); FDA, Food and Drug Administration.

Therefore, the International Myeloma Working Group (IMWG) recently revised the response criteria and introduced the definition of MRD in CR patients as the persistence or re-emergence of very low levels of cancer cells, equal to about 1 tumor cell in at least 10^5^ normal cells (3). These response criteria are the direct result of the progressive evolution of both imaging and bone marrow MRD techniques in the last 15 years (Figure 1). However, a precise knowledge of when and how to perform MRD detection is required. This review aims to examine the currently available MRD techniques recommended by IMWG and data from different clinical trials, in order to outline a possible future perspective on the role of MRD testing as a tool for decision making in standard clinical practice.

## MRD Techniques and Practical Considerations

### Bone Marrow Techniques: NGF and NGS

There are two techniques commonly used to detect MRD in the bone marrow (BM): multiparameter flow cytometry (MFC) and next-generation sequencing (NGS) molecular technology. Both techniques show positive and negative aspects (Table 1).

**Table 1:** Bone marrow techniques for MRD in myeloma: pros and cons.

	**Next-generation flow (NGF)**	**Next-generation sequencing (NGS)**
Applicability	Nearly 100%	≥90%
Availability	Many laboratories with 4–6 colors; >8 colors restricted to more specialized centers	Commercial service only; ongoing efforts by academic platforms
Diagnostic sample	Not required	Required for identification of dominant clonotype
Number of cells required	10 million cells/tube	1–2 million cells/20 μg DNA
Sample processing	Requires a fresh sample; assessment within 24–48 h	Can use both fresh and stored samples
Standardization	EuroFlow consortium	Commercial companies (Adaptive Biothcnologies) Academic methodologies also available
Sample quality control	Possible to check by global bone marrow cell analysis	Not possible
Quantitative	Yes	Yes
Sensitivity	1 in 10^−5^-10^−6^	1 in 10^−5^-10^−6^
Turnaround and complexity	3–4 h. Requires flow cytometry skills. Automated software available	1 week. Academic methodologies require bioinformatics support
Clonal evolution	Not evaluable	Evaluable: can take into account all minor clones

*MRD, minimal residual disease*.

MFC can detect and quantify tumor vs. normal plasma cells using cell surface and cytoplasmic markers. For the identification of plasma cells, the combined use of CD38 and CD138 is recommended even if they are also expressed on other BM cells. In particular, the aberrant expression patterns of CD19, CD56, CD45, CD38, CD27, CD20, CD28, CD33, CD117 and surface membrane immunoglobulin can characterize the phenotype of monoclonal plasma cells (4). However, antigenic expression can vary on plasma cells and should be considered when interpreting flow data.

Older conventional 4- to 7-color flow cytometry assays have now been replaced by advanced 8-color 2-tube or 10-color 1-tube assays. In this sense, the increased sensitivity of MFC (between 10^−4^ and 10^−5^) is due to the simultaneous assessment of ≥8 markers in a single tube. In this way, if sufficient cell numbers are evaluated (e.g., ≥5 × 10^6^), it is possible to promptly identify aberrant PC phenotypes at MRD levels (5).

A consensus methodology has been recently proposed by the International Myeloma Foundation's Black Swan Research Initiative, which formed the EuroFlow Next-Generation Flow (NGF) panel in order to increase sensitivity and standardization of MFC (6, 7). This panel includes two 8-color tubes (tube 1: CD138, CD27, CD38, CD56, CD45, CD19, CD117, CD81; tube 2: CD138, CD27, CD38, CD56, CD45, CD19, cIgκ, cIgλ). In this protocol, a bulk-lysis procedure was determined and the acquisition of ≥10^7^ cells/sample was recommended. Importantly, new softwares were developed for automatic plasma cell gating in order to avoid individual assessments.

According to this consensus methodology, it is important to evaluate the limit of quantitation (LOQ) and the limit of detection (LOD) of the NGF-MRD method. The LOQ is calculated as 50 among 10^7^ nucleated cells (based on the identification of ≥50 clonal plasma cells); the LOD as 20 among 10^7^ nucleated cells (based on the identification of ≥20 clonal plasma cells). This evaluation allows to discriminate between positive and negative samples. Interestingly, a baseline sample is not mandatory for MRD evaluation. After the multicenter evaluation of patients with very good partial response (VGPR) or CR, 110 follow-up bone marrows showed a higher sensitivity for NGF-MRD, as compared to conventional 8-color flow-MRD: MRD-positive rates were 47 vs. 34% (*P* = 0.003), respectively. Thus, 25% of patients who were categorized as MRD-negative by conventional 8-color flow were categorized as MRD-positive by NGF. This translated into a significantly longer PFS using NGF to discriminate between MRD-negative and MRD-positive CR patients (*P* = 0.02). Importantly, NGF can also provide a qualitative assessment of the patient sample by allowing the complete analysis of the normal B-cell compartment and the detection of a significantly decreased number of non-PC BM cells (e.g., mast cells, nucleated red blood cells, myeloid precursors, B-cell precursors, and CD19– normal PC) revealing potentially hemodiluted BM samples. Finally, treatment with CD38 antibodies such as daratumumab and isatuximab can alter the antigen expression in MM cells. This sets a limit for the use of CD38 as a marker for the detection of plasma cells during MRD assessments at follow-up. The use of multi-epitope CD38 antibody in an advanced flow cytometry panel can solve this problem, since this conjugate can bind to a specific site (not covered by daratumumab) of the CD38 antigen. Nonetheless, in case of CD38 surface downregulation, the solution is the analysis of intracellular CD38 through the same protocol used for intracellular k- and λ-chain staining (7).

Allele-specific oligonucleotide polymerase chain reaction (ASO-PCR) was first explored to evaluate molecular MRD in MM, but even if its prognostic role was confirmed, different issues limited its use in favor of the NGS technique. First, its applicability ranged from 40 to 60% due to the low rate of diagnostic marker identification, since this technique does not take into account the somatic hypermutation rate of immunoglobulin loci and this translates into sequencing problems. Moreover, patient-specific reagents raised the complexity of this technique (8–11).

NGS was developed to overcome all these disadvantages. ClonoSEQ® Assay (Adaptive Biotechnologies, Seattle, US-WA) is the most frequently adopted commercial platform in the United States. In this test, DNA is extracted from patient's BM, a multiplex PCR amplifies VDJ, IgK, and IgL gene sequences and a common PCR prepares DNA for sequencing and creates a sequencing library. At the end of the process, a bioinformatic tool is essential to extrapolate and analyze all NGS data.

Using this assay, we can define as “clonotypes” two identical sequencing reads. A clonotype with frequency >5% at diagnosis is considered a clonality (clonal gene rearrangements), thus becoming a target for the detection of MRD in follow-up samples (12, 13). In lymphoid malignancies, NGS and ASO-PCR have been compared, showing similar sensitivities and results (13).

In the IFM2009 clinical trial, a comparison between NGS and 7-color MFC has been made, showing that the higher sensitivity with NGS at 10^−6^ allowed to predict the best outcomes in MRD-positive vs. -negative patients (3-year PFS: 53 vs. 83%, *p* < 0.001).

Ongoing clinical trials are evaluating NGS vs. NGF and their correlation: in the CASSIOPEIA trial, a good concordance (83.5% in paired samples) was observed using the same sensitivity (10^−5^) regardless of response in patients achieving ≥CR, indicating that both techniques performed similarly in evaluating MRD (14). As illustrated in Table 1, some characteristics can affect the clinician's preference of choosing NGS vs. NGF, such as the higher cost for NGS (~1,500 $ per sample vs. ~300 $ for NGF), and the required time and skills (at least 1 week for NGS vs. 3–4 h for NGF and commercial service available only for NGS).

In this regard, ongoing studies are evaluating ‘in-house’ NGS techniques: recently, Martinez-Lopez et al. described a NGS method starting from 1 μg of DNA and amplified IGH or IGK sequences. The sequencing data were analyzed by specific mathematical and bioinformatic tools to identify and quantify the clonotype present on each sample. A clonotype was identified when at least 400 identical sequencing reads were obtained, or when it was present at a frequency of >1% with a sensitivity of at least 10^−5^ (15).

### Imaging Techniques: PET/CT

MM is a patchy disease and BM infiltration may often be heterogeneous. Indeed, ~60% of MM patients show focal lesions that represent the local accumulation of plasma cells (16). Therefore, the IMWG incorporated imaging in addition to BM evaluation to better characterize MM residual disease (3).

Different studies showed the role of imaging techniques in evaluating focal lesions: magnetic resonance imaging (MRI) is a sensitive, non-invasive imaging technique available to detect the bone involvement in the spine and to provide details regarding the soft tissue disease and the pattern of marrow infiltration (normal, focal, diffuse, or heterogeneous).

Fluorodeoxyglucose positron emission tomography/computed tomography (FDG PET/CT) can be used to analyze the vitality of the focal lesions and is therefore the current standard of care to evaluate the post-therapeutic residual infiltration (17–19).

Different studies showed the prognostic and predictive role of FDG PET/CT (20–22). Interestingly, Moreau et al. compared PET/CT with MRI. Although at diagnosis both the techniques performed similarly in the detection of bone lesions, the normalization after therapy of PET/CT, but not of MRI, was predictive of PFS and OS (20). In both responding and non-responding patients, focal lesions can still remain positive for many months. As a consequence, conventional MRI is probably not the best technique to evaluate MRD (22–24). On the other hand, functional MRI techniques based on the measurement of the movement of water molecules in the tissue (Diffusion-Weighted MRI, DWI) could be informative on the residual cellularity and the microcirculation of the focal lesions (25). No standardization of the diagnostic technique and no interpretation of results in MM after therapy are still available and no prospective comparison between PET/CT and DWI in a meaningful number of patients has been done. In a small number of MM patients, DWI seemed to be more sensitive in the detection of residual lesions. However, if this could be an advantage or could lead to an increased number of false-positive cases, still needs to be elucidated (26, 27).

Finally, different researchers confirmed the complementarity of PET/CT and BM techniques. Rasche et al. showed how patients who were both Flow-MRD- and PET/CT-negative had the best PFS outcome when compared with those who were Flow-MRD-negative but PET/CT-positive (28). Paiva et al. demonstrated that, even if NGF-negative patients had a long PFS, there was a proportion of subjects who relapsed with extramedullary disease in the presence of a previous negative BM sample, confirming the importance of combining BM and imaging analyses (29).

PET/CT has some limitations, some of which are linked to the tracer used (FDG). Indeed, a low expression of the enzymes responsible for the glycolysis process (e.g., hexokinase 2 gene) in MM cells could lead to false-negative cases with FDG PET/CT (30). Alternative tracers could overcome these limitations. For instance, 11C-Methionine uptake correlates with protein synthesis, a very active mechanism in malignant plasma cells, and can be used as an alternative PET/CT tracer in MM (31).

In a head-to-head prospective comparison in a heterogeneous MM patient population, 11C-Methionine PET/CT was more sensitive than FDG PET/CT in the detection of focal lesions, both within and outside the bone. More data are needed in a homogenous patient population to understand whether this tracer could be an alternative to FDG in the detection of residual disease after treatment. Currently, other tracers targeting lipid membrane (e.g., Choline, Acetate) and CXCR4 are also under study (32).

## MRD Results in the Clinical Setting: Relevant Questions

In this section we focus on clinically relevant questions regarding MRD, reviewing available data on newly diagnosed MM (NDMM) patients. Single studies are summarized in Table 2. Data on MRD evaluation in relapsed and/or refractory MM patients (59) and high-risk smoldering MM (60) are beginning to emerge as well, and have been recently reviewed elsewhere (61).

**Table 2:** Selected trials on NDMM patients reporting MRD data.

**Study**	**Patient population^a^ (*n*)**	**Method (sensitivity)**	**Timepoint**	**MRD-negative^b^ (%)**	**Outcomes (MRD neg vs. MRD pos)**
Paiva et al. (33)	TE NDMM in ≥PR after 6 alternating VBMCP/VBAD cycles and ASCT (295)	MFC (10^−4^)	+100 days after ASCT	42%	Median PFS: 71 vs. 38 months
Rawstron et al. (34)	NDMM: - Intensive arm (378): CTD (178) or CVAD (190) induction + ASCT. - Non–intensive arm (245): MP (119) or aCTD (126).	MFC (10^−4^)	Post-induction (both arms) and +100 days after ASCT (intensive arm only)	Intensive arm: Post CTd induction 25% (71% post-ASCT) Post CVAD 13% (54% post-ASCT) Non-intensive arm: Post MP induction 3% Post aCTd induction 26%	Intensive arm: Median PFS^*^ according to post-ASCT timepoint 29 vs. 16 months Non-intensive arm: Median PFS^*^ according to post-induction timepoint 10.5 vs. 7.4 months
Puig et al. (10)	NDMM in ≥PR (102)	ASO-PCR (10^−4^)	Post-induction (NTE patients) or +100 days after ASCT (TE patients)	46%	TE patients: median PFS 54 vs. 27 months NTE patients: median PFS NR vs. 31 months
Kumar et al. (35)	NDMM receiving IRd induction + ixazomib maintenance (64)	MFC (10^−4^)	Mostly at suspected CR	12.5%	NA
de Tute at al. (36)	NTE NDMM after aCTD or aRCD induction (297)	MFC (10^−4^)	Post-induction	aCTD arm: 11% aRCD arm: 16%	aCTD arm: median PFS 34 vs. 19 months aRCD arm: median PFS 32 vs. 17 months
Ludwig et al. (37)	TE NDMM in CR after 4 cycles of VTd or VTd+cyclophosphamide induction and ASCT (42)	MFC (not specified)	Suspected CR	81%	Median PFS NR vs. 39 months
Paiva et al. (38)	NTE NDMM in ≥PR after 6 VMP (52) or VTP (50) induction cycles	MFC (10^−4^-10^−5^)	Post-induction	30%	3-year PFS: 90% vs. NR
Roussel et al. (39)	TE NDMM after 3 VRd + ASCT + 2 VRd cycles followed by lenalidomide maintenance (31)	MFC (10^−4^-10^−5^)	Longitudinal	Post-induction: 16% Post-ASCT: 54% Post-consolidation: 58% Post-maintenance: 68%	3-year PFS according to post-maintenance MRD: 100% vs. 23%
Paiva et al. (40)	TE NDMM in ≥CR after ASCT (241)	MFC (10^−4^-10^−5^)	+100 days after ASCT	64%	3-year TTP: 76% vs. 58%
Ferrero et al. (41)	TE NDMM in ≥VGPR after ASCT (39) undergoing VTd consolidation	ASO-PCR (10^−4^-10^−5^)	Longitudinal	Post-ASCT: 23%, Post-consolidation: 57% 6-month post-consolidation: 72%	Median PFS: 68 vs. 23 months
Korthals et al. (11)	TE NDMM after 2–4 cycles of idarubicin-dexamethasone undergoing ASCT	ASO-PCR (10^−4^-10^−5^)	Post-induction and post-ASCT (+3–6 months)	Post-induction: 17% Post-ASCT: 21%	NA
Lahuerta et al. (42)	NDMM alive and with MRD data available at 9 months after treatment start (609)	MFC (10^−4^-10^−5^)	9 months after treatment start	43%	Median PFS^*^ 63 months vs. NA (11–29 months in the other response categories)
Gu et al. (43)	TE NDMM (101)	MFC (50^−4^-10^−5^)	Longitudinal	Post-induction: 37% Post-ASCT: 66% 2-year post-ASCT: 78%	Median TTP: NR vs. NR
Korde et al. (44)	NDMM receiving 8 KRd induction cycles (45)	NGS (not specified)	Post-induction	42% (calculated on NGS-evaluable NDMM patients)	18-month PFS: 100% vs. 84%
Martin-Lopez et al. (45)	NDMM in ≥VGPR (121)	NGS [10^−5^]	Post-induction (NTE patients) or +100 days after ASCT (TE patients)	27%	Median TTP: 80 vs. 31 months
Oliva et al. (46)	TE NDMM in ≥VGPR after consolidation (73) followed by lenalidomide maintenance	ASO-PCR (10^−5^)	Pre-maintenance and during maintenance	Pre-maintenance: 45% During maintenance: 60%	Median PFS: NR vs. 48 months
Oliva et al. (47)	TE NDMM in ≥VGPR after VCd induction, VMP vs. ASCT intensification, VRd vs. no consolidation (316) followed by lenalidomide maintenance	MFC (10^−5^)	Pre-maintenance and during maintenance	Post-consolidation: 76%	3-year PFS^*^: 77% vs. 50%
Paiva et al. (48)	NTE NDMM with response (80% of the patients with ≥VGPR) after 18 sequential or alternating VMP/Rd cycles (162)	MFC (10^−5^)	After 9 cycles or 18 cycles	Sequential arm 9-cycles: 20% 18-cycles: 46% Alternating arm 9-cycles: 19% 18-cycles: 33%	Median TTP^*^: NR vs. 15 months
Mateos et al. (49)	NTE NDMM: DaraVMp arm (350) - VMp arm (356)	NGS (10^−5^)	Longitudinal	- Dara-VMp arm: 22.3% - VMp arm: 6.2%	NA
Facon et al. (50)	NTE NDMM: - DaraRd arm (368) - Rd arm (369)	NGS (10^−5^)	Longitudinal	Dara-Rd: 24.2% Rd arm: 7.3%	NA
Voorhees et al. (51)	TE NDMM receiving Dara-VRd induction, ASCT and Dara-VRd consolidation (13)	NGS (10^−5^)	Longitudinal	Post-induction: 19% Post-consolidation: 50%	NA
Gay et al. (52)	TE NDMM receiving KCd-ASCT-KCd (arm A, 159), KRd-ASCT-KRd (arm B, 158), 12 cycles of KRd (arm C, 157)	MFC (10^−5^)	Pre-maintenance	Arm A: 42% Arm B: 58% Arm C: 54%	NA
Flores-Montero et al. (7)	NDMM or RRMM patients achieving ≥VGPR (79)	NGF (10^−5^-10^−6^)	Post-induction, during maintenance or post-treatment	47%	Time to 75% PFS event^*^: NR vs. 10 months
Hahn et al. (53)	NDMM receiving induction and ASCT ± VRd consolidation (293) followed by lenalidomide maintenance	MFC (10^−5^-10^−6^)	Longitudinal	Pre-ASCT 42% Post-ASCT ± consolidation 78% 1 year post-ASCT 84%	Pre-ASCT 3-year PFS^*^: 69% vs. 60% Post-ASCT ± consolidation 3-year PFS^*^: 75 vs. 59% 1-year post-ASCT 3-year PFS^*^: 76% vs. 44%
Ocio et al. (54)	NTE NDMM receiving Isa-VRd induction + Isa-Rd maintenance (16)	NGF (10^−5^) and NGS (10^−5^)	Longitudinal	NGF 44% (18% at 10^−6^) NGS 50% (33% at 10^−6^)	NA
Zimmermann et al. (55)	TE NDMM receiving 4 cycles of KRd induction-ASCT-4 cycles of KRd consolidation and 10 cycles of KRd extended consolidation (76)	MFC (10^−4^-10^−5^) and NGS (10^−6^)	Longitudinal	MFC Post-consolidation (cycle 8) 82% Post-extended consolidation (cycle 18) 90% NGS Post-consolidation (cycle 8) 66% Post-extended consolidation (cycle 18) 71%	According to cycle 8 MRD status by MFC and/or NGS 2-year PFS: 100 vs. 93%
Avet-Loiseau et al. (56)	NDMM receiving DaraVTd-ASCT-DaraVTd (543) or VTd-ASCT-VTd (542)	MFC (10^−5^) and NGS (10^−6^)	Post-induction Post-consolidation	Post-induction (MFC) Dara-VTd arm: 35% VTd arm 23% Post-consolidation (MFC) Dara-VTd arm: 64% VTd arm 44% Post-consolidation (NGS in evaluable patients) Dara-VTd arm: 39% VTd arm 23%	NA
Takamatsu et al. (57)	NDMM in ≥VGPR after ASCT (51)	NGS (10^−6^)	Post-ASCT (day 24–2,808)	51%	4-year PFS: 96% vs. NR
Perrot et al. (58)	TE NDMM after 8 VRd cycles or 3 VRd + ASCT + 2 VRd cycles followed by lenalidomide maintenance (509)	NGS (10^−6^)	Pre- or post-maintenance	VRd alone arm: 20% ASCT arm: 30%	Median PFS: NR vs. 29 months

a *If data come from a heterogeneously treated population, information about treatment is not showed. If data come from a single randomized trial, treatment data are provided*.

b *If data at different sensitivity levels are available, the MRD rates at highest sensitivity levels are provided*.

* *time-to-event calculated from MRD assessment*.

*NDMM, newly diagnosed multiple myeloma; MRD, minimal residual disease; CR, complete response; VGPR, very good partial response; MFC, multiparametric flow cytometry; ASCT, autologous stem-cell transplantation; TTP, time-to-progression; PFS, progression-free survival; ASO-PCR, allele-specific oligonucleotide polymerase chain reaction; TE, transplant-eligible; NGS, next-generation sequencing; NR, not reached; NA, not available; NTE, transplant-ineligible; Dara, daratumumab; Ixa, ixazomib; Rd, lenalidomide, dexamethasone; VRd, bortezomib, lenalidomide, dexamethasone; IRd, ixazomib, lenalidomide, dexamethasone; VTd, bortezomib, thalidomide, dexamethasone; VMP, bortezomib, melphalan, prednisone; VCd; bortezomib, cyclophosphamide, dexamethasone; KRd, carfilzomib, lenalidomide, dexamethasone; KCd, carfilzomib, cyclophosphamide, dexamethasone; CRd, cyclophosphamide, lenalidomide, dexamethasone; PR, partial response; VBMCP, vincristine, carmustine, melphalan, cyclophosphamide, prednisone; VBAD, vincristine, carmustine, adriamycin, dexamethasone; CTD, cyclophosphamide, thalidomide, dexamethasone; RCD, lenalidomide, cyclophosphamide, dexamethasone; CVAD, cyclophosphamide, vincristine, doxorubicin, dexamethasone; MP, melphalan and prednisolone; aCTD/aRCD, attenuated CTD/RCD; NGF, next-generation flow*.

In the MM field, a major question concerned the prognostic role of MRD and its ability to perform better than conventionally defined response criteria. As already discussed, there is now compelling evidence coming from multiple studies (Table 2) and two meta-analyses (2, 62) confirming that MRD-negative patients have a significantly better PFS and OS compared to MRD-positive patients. The beneficial effect of MRD negativity was confirmed also focusing on CR patients (2). Using MFC with a sensitivity of 10^−4^-10^−5^, Lahuerta et al. nicely demonstrated that MRD-negative patients with a conventionally defined CR had better PFS (median, 63 vs. 27 months, *p* < 0.001) and OS (median, not reached vs. 59 months, *p* < 0.001) than MRD-positive CR patients (42). Moreover, MRD-positive CR patients had similar outcomes compared to patients achieving a partial response (PR) (median PFS, 27 vs. 29 months; median OS, 59 vs. 65 months, respectively) showing that the prognostic advantage of conventionally defined CR over PR resided in the MRD-negative patient population (42).

The best timing for MRD measurement is another important unanswered question. Usually, MRD is measured at specific timepoints during therapy [e.g., *post-induction* (39), +*100 days post-ASCT* (33), *post-consolidation* (41), *pre-maintenance*, and *during maintenance* (46)]. If treatment does not provide for a phase-specific timepoint (as in the case of the continuous treatment strategy commonly adopted for transplant-ineligible patients), MRD testing is usually done at unconfirmed CR/sCR and at fixed timepoints thereafter (50).

Data clearly show that, as we continue to intensify patient treatment, the percentage of MRD-negative patients increases (39, 43, 53, 55, 56) and even maintenance treatment can convert a significant percentage of MRD-positive patients into MRD-negative [e.g., 27–30% with lenalidomide maintenance in a pooled analysis (9, 46)]. Each timepoint can be important due to different clinical reasons. For instance, the *post-induction* timepoint can be used to design clinical trials addressing different intensification regimens, while *pre-maintenance* or *during maintenance* timepoints can be exploited to design clinical trials addressing the intensity and the duration of maintenance. Regarding the prognostic effect of different timepoints, in the Myeloma IX study, which used MFC with a sensitivity of 10^−4^, a PFS advantage was found in patients that were MRD-negative both *post-induction* and *post-ASCT*, as compared with patients that were MRD-positive *post-induction* and became MRD-negative *post-ASCT*, although this effect did not translate into an OS benefit (34). On the other hand, Hahn et al. demonstrated in a transplant-eligible population that patients who were MRD-negative *pre-ASCT, pre-maintenance*, and *1-year post-ASCT* showed all a better PFS compared to MRD-positive patients. Only the *1-year post-ASCT* timepoint was associated with better OS (3-year post-ASCT OS 96 vs. 66% for MRD-negative vs. MRD-positive patients) (53). These data suggest that the duration of MRD negativity may be important, but little data are available on sustained MRD negativity (i.e., the need to confirm MRD at different timepoints) and on its optimal duration. Gu et al. used MFC to monitor 104 MM patients *post-induction* and at different *post-ASCT* timepoints (3 to 24 months), showing that patients with persistent MRD negativity *post-induction* until 24 months after ASCT (*n* = 33) had better time to progression (median, not reached vs. 15.4 months) and OS (not reached vs. 35.2 months), as compared to patients that were MRD-negative *post-induction* but MRD-positive within 24 months *post-ASCT* (*n* = 5) (43). The low numbers in the latter group do not allow the exploration of different time cutoffs for sustained MRD negativity. However, 2/5 patients became MRD-positive 18 months *post-ASCT*, thus suggesting that long-term confirmation of sustained MRD negativity may be necessary.

Another question is whether the sensibility of the technique impacts the reliability of MRD. Using MFC with a sensibility of 10^−4^, Rawstron et al. demonstrated that each log depletion in MRD levels predicted a 1-year median OS advantage (5.9 years for 10^−2^-10^−3^, 6.8 years for 10^−3^-10^−4^, and more than 7.5 years for 10^−4^), suggesting that MRD level is a continuous rather than a discrete variable (63). Recently, several studies using both flow cytometry-based methods with a sensitivity of 10^−5^ (48) or 10^−5^-10^−6^ (7) and NGS-based methods with a sensitivity of 10^−6^ (58, 64) demonstrated that lower levels of MRD are associated with better outcomes and that the best possible sensitivity should be pursued. Indeed, in the IFM/DFCI 2009 trial, among 163 patients who were MRD-negative *pre-maintenance* using MFC with a sensibility of 10^−4^, 84 (56%) were indeed MRD-positive using NGS with a sensibility of 10^−6^ (3-year PFS, 86 vs. 66% in NGS-negative vs. NGS-positive among MFC-negative patients). This is especially important in clinical trials designed to explore treatment interruption based on MRD levels because a low sensibility of the technique can lead to unacceptable risk of patients' undertreatment.

This observation leads to our last question: if MRD negativity is a major prognostic determinant, do treatment administered and baseline risk stratification matter as long as MRD negativity is achieved? Many studies demonstrated that even if a more effective regimen induced MRD negativity in a higher number of patients, the prognosis of MRD-negative patients was similar independently from treatment arm (49, 58). However, we do need MRD-driven clinical trials to determine if treatment deintensification in MRD-negative patients is feasible without worsening patient prognosis (65). In this regard, in the Myeloma IX trial, MRD-negative patients (MFC at 10^−4^) receiving thalidomide maintenance remained in a MRD-negative state more often than patients not receiving maintenance treatment (96 vs. 68.8%, *p* = 0.026). Regarding MM patients who are at high risk according to baseline prognostic factors (e.g., high-risk cytogenetics or unfavorable Revised International Staging System score), MRD-negative patients at a low level of sensitivity (10^−4^) still showed inferior clinical outcomes than standard-risk patients (34). Conversely, reaching MRD negativity at a sensitivity of 10^−5^-10^−6^ seemed to overcome the inferior outcome observed in high-risk vs. standard-risk patients (48, 58). However, it should be noted that high-risk patients require highly intensive regimens in order to achieve a proper level of MRD negativity (47, 52, 55).

## Future Perspectives

### Is MRD a Surrogate Endpoint for Drug Approval?

Improving OS and quality of life is the final aim of MM treatment. In the past years, the PFS endpoint has been used as a surrogate endpoint for OS to speed up the drug approval process. However, following the achievement of long-standing and deep responses (especially in NDMM patients), PFS is inappropriately becoming a late endpoint. MRD is considered the best candidate as a PFS/OS surrogate marker for provisional drug approval by regulatory agencies. Indeed, ClonoSEQ® Assay is now authorized by FDA (66) and MRD negativity with a sensitivity of 10^−5^ is the most common primary endpoint of new clinical trials designed for NDMM patients. However, as discussed above, continuous efforts should be exterted to define the optimal sensitivity cut-off (10^−5^ vs. 10^−6^), the timing of evaluation and the need for a sustained MRD negativity. Moreover, safety should be closely addressed, as it was demonstrated by higher MRD (13.4 vs. 1%) but worse OS rates (HR 2.03, 95% CI 1.04–3.94) in the experimental arm of the BELLINI trial (M14-031) comparing venetoclax-Vd vs. Vd (67, 68). Moreover, in some settings, the correlation between MRD negativity rates and PFS improvement could be less clear because of technical pitfalls (e.g., early MRD evaluation after myelosuppressive treatments in hypocellular bone marrows).

#### How to Address Spatial Heterogeneity?

MM is a spatially heterogeneous disease and simultaneous MRD negativization inside and outside the bone marrow showed synergistic predictive values (28).

Moreover, MRD analysis within the bone marrow is done on bone marrow aspirates coming from a single random site and, in some patients, MM cells show a patchy infiltration (69). To overcome this issue and to possibly link the information on residual disease coming from both bone marrow and extramedullary sites, liquid biopsy approaches are beginning to emerge. Currently under exploration are the detections at high sensitivity levels of circulating tumor DNA (70), circulating plasma cells (71), and M protein peptides (72–74). The further optimization of the available techniques will be essential for their future success.

As an example, applying the ClonoSEQ® assay to peripheral blood ctDNA and paired BM samples, Mazzotti et al. showed that residual disease in the peripheral blood was undetectable in 69% of patients with concurrent MRD-positive bone marrow samples (70). This was mainly due to an insufficient sensibility to detect specific Ig gene rearrangements in the peripheral blood when disease burden was low in the BM (70), underlying the need to improve the technique before we can routinely exploit peripheral blood to monitor MM burden.

#### MRD-Driven Trials

MRD has not yet entered the clinical practice, but it represents an attractive tool to potentially guide treatment choices. To address this hypothesis, many MRD-driven trials are beginning to explore treatment intensification in MRD-positive patients after standard treatment (e.g., NCT03901963) or treatment deintensification in sustained MRD-negative patients (e.g., NCT03710603). Ongoing and future MRD-driven trials will contribute to solve the unanswered question: is it recommended to evaluate other induction cycles until the achievement of MRD negativity in patients who are MRD-positive after 4 induction cycles? Can we perform post-transplant consolidation on the basis of MRD status? Can we stop maintenance after 1 year of sustained MRD negativity?

Ongoing and future clinical trials will evaluate the definition and the role of sustained MRD-negativity in treatment decision-making. On the one hand, the achievement of a MRD-negative status does not necessarily mean that treatment should be stopped. Indeed, it should be noted that what we define as “MRD-negative” is a MRD undetectable with the current techniques, each one of them having a sensitivity limit. This means that we are not sure that the disease is eradicated even in MRD-negative cases. On the other hand, the achievement of a MRD-positive status after treatment brings the question of whether it is necessary to change treatment, improving the depth of response. However, before developing response-adjusted treatment strategies based on MRD status—either intensifying/changing treatment for MRD-positive patients or de-escalating treatment for MRD-negative patients—we need to understand if sustained MRD negativity should be the treatment goal and to define the most appropriate timepoint for its evaluation (after 1 year or after more years).

## Author Contributions

SO, MD'A, MB, and AL: substantial contributions to the conception or design, acquisition, analysis, or interpretation of data, critical revision for important intellectual content, final approval of the version to be published, and agreement to be accountable for all aspects of the work in ensuring that questions related to the accuracy or integrity of any part of the work are appropriately investigated and resolved. SO, MD'A, and AL: first draft. MB and AL: supervision.

### Conflict of Interest

SO has received honoraria from Amgen, Celgene, and Janssen; has served on the advisory boards for Adaptive Biotechnologies, Janssen, Amgen, and Takeda. MD'A has served on the advisory board for GSK. MB has received honoraria from Sanofi, Celgene, Amgen, Janssen, Novartis, AbbVie, and Bristol-Myers Squibb; has received research funding from Celgene, Janssen, Amgen, Bristol-Myers Squibb, Mundipharma, Novartis, and Sanofi. AL has received honoraria from Amgen, Bristol-Myers Squibb, Celgene, and Janssen; has served on the advisory boards for Bristol-Myers Squibb, Celgene, Janssen, and Takeda.
